# A Single Day of Excessive Dietary Fat Intake Reduces Whole-Body Insulin Sensitivity: The Metabolic Consequence of Binge Eating

**DOI:** 10.3390/nu9080818

**Published:** 2017-07-29

**Authors:** Siôn A. Parry, Rachel M. Woods, Leanne Hodson, Carl J. Hulston

**Affiliations:** 1 School of Sport, Exercise and Health Sciences, Loughborough University, Loughborough, Leicestershire LE11 3TU, UK; S.Parry@lboro.ac.uk (S.A.P.); R.M.Woods@lboro.ac.uk (R.M.W.); 2 Oxford Centre for Diabetes, Endocrinology and Metabolism, University of Oxford, Churchill Hospital, Oxford OX3 7LE, UK; leanne.hodson@ocdem.ox.ac.uk

**Keywords:** glucose, insulin, high-fat, overfeeding, glycemic control, type 2 diabetes mellitus

## Abstract

Consuming excessive amounts of energy as dietary fat for several days or weeks can impair glycemic control and reduce insulin sensitivity in healthy adults. However, individuals who demonstrate binge eating behavior overconsume for much shorter periods of time; the metabolic consequences of such behavior remain unknown. The aim of this study was to determine the effect of a single day of high-fat overfeeding on whole-body insulin sensitivity. Fifteen young, healthy adults underwent an oral glucose tolerance test before and after consuming a high-fat (68% of total energy), high-energy (78% greater than daily requirements) diet for one day. Fasting and postprandial plasma concentrations of glucose, insulin, non-esterified fatty acids, and triglyceride were measured and the Matsuda insulin sensitivity index was calculated. One day of high-fat overfeeding increased postprandial glucose area under the curve (AUC) by 17.1% (*p* < 0.0001) and insulin AUC by 16.4% (*p* = 0.007). Whole-body insulin sensitivity decreased by 28% (*p* = 0.001). In conclusion, a single day of high-fat, overfeeding impaired whole-body insulin sensitivity in young, healthy adults. This highlights the rapidity with which excessive consumption of calories through high-fat food can impair glucose metabolism, and suggests that acute binge eating may have immediate metabolic health consequences for the individual.

## 1. Introduction

Binge eating, which is defined as discreet periods of excessive food consumption that is not driven by hunger or metabolic need [1,2], is strongly associated with overweight and obesity [3,4,5], which are risk factors for developing insulin resistance and the metabolic syndrome [6]. Those who exhibit binge eating behavior are known to eat until they are uncomfortably full and then may or may not compensate for this increased energy intake, with the latter leading to a positive energy balance [1]. The etiology of binge eating and binge eating disorders (BED: the clinical manifestation of binge eating behavior [7]) has been extensively studied from a psychological perspective, with findings indicating that a number of stressors (e.g., interpersonal, ego-threatening, and work-related) are associated with increased food intake and inter-meal snacking [8,9]. However, the metabolic response to such behavior has received relatively little attention and, therefore, little is known regarding the metabolic consequences.

Binge eating has been associated with a selective increase in the intake of palatable foods (i.e., foods which are high in fat and/or sugar) [7]. This is important, as dietary composition appears to be a key mediating feature in the pathogenesis of metabolic disease. For example, high-fat diets or the consumption of saturated fatty acid (SFA)-enriched diets have been associated with insulin resistance [10,11]. Results from the Kuopio, Aarhus, Naples, Wollongong and Uppsala (KANWU) study demonstrated that consumption of an SFA-rich diet for three months reduced insulin sensitivity in healthy adults when compared to a diet enriched in monounsaturated fatty acids (MUFA) [12]. Furthermore, when stratified by habitual total fat intake (above and below median), individuals with a higher fat intake (>37% total energy (TE)) exhibited reduced insulin sensitivity compared to those with a low intake (<37% TE) [13]. The mechanism by which a high total fat or SFA-diet causes insulin resistance remains unclear. One hypothesis is that adipose tissue dysfunction leads to an overabundance of circulating non-esterified fatty acid (NEFA) and insulin resistance ensues due to the accumulation of NEFA in non-adipose tissue organs, such as skeletal muscle and the liver [14]. Although work from the 1960s [15,16] supports the hypothesis as elevated NEFA concentrations were observed in obesity; recent reports are not in agreement [17]. Experimentally elevating plasma NEFA concentrations, via intravenous (iv) infusion of a lipid-heparin emulsion (Intralipid), rapidly (within 6–8 h) reduces insulin-mediated glucose disposal in healthy, lean individuals [18,19,20]. This is associated with a sequential pattern of events starting with an increase in circulating triglyceride (TG) levels (<0.5 h), followed by a supraphysiological increase in circulating NEFA levels (~1–1.5 h), a rise in intramyocellular lipid (IMCL) content (~2.5 h), and finally a reduction in insulin sensitivity; this supports the concept that elevated NEFA levels are a key player in the development of insulin resistance. However, during iv lipid infusions, plasma NEFA concentrations are typically elevated to around 1500 μmol/L or higher [17], which is in excess of the fasting concentrations reported for obese, insulin-resistant individuals (~400 µmol/L) [21], and individuals with poorly managed type 2 diabetes mellitus (T2DM) (~800 µmol/L) [22].

Results from short-term (4–14 days), high-fat overfeeding interventions report impaired glycemic control [23,24,25] and decreased hepatic [26] and whole-body insulin sensitivity [27,28] despite unchanged or reduced plasma NEFA concentrations. Although these studies demonstrate that impairments in glycemic control occur after several days or weeks of consistent overconsumption, this model may not reflect the dietary practices of those who binge eat and consume a severe energy excess within a matter of hours. Although it remains unclear if episodes of binge eating have a negative impact on glucose metabolism, there is some evidence, albeit limited, to suggest that diet-induced impairments may occur very rapidly. Nowotny et al. [29] reported that oral administration of a single dose of soybean oil (100 mL), which is enriched with polyunsaturated fat (61% polyunsaturated, 23% monounsaturated, and 16% saturated), reduced whole-body insulin sensitivity (assessed by hyperinsulinemic-euglycemic clamp) to a comparable extent and within a similar time-frame (6 h post ingestion/infusion) as an energy and composition-matched iv lipid-heparin infusion. This occurred independent of plasma NEFA levels, which were elevated during the iv fat infusion but were unchanged following oral fat ingestion [29]. Insulin sensitivity was assessed 6–8 h after fat ingestion/infusion [29], and it is possible that the observed reduction in insulin sensitivity was a transient response related to the ongoing metabolism of fat; it would be of interest to determine if changes persist into the post-absorptive state and occur after the consumption of a diet more reflective of Western style eating patterns (i.e., SFA rather than polyunsaturated fatty acid (PUFA)-enriched). Therefore, in order to replicate excessive binge eating behavior, we undertook a pilot study in which whole-body insulin sensitivity was assessed in young, healthy, non-obese individuals after a single day of high-energy, high-fat, SFA-rich food intake.

## 2. Materials and Methods

### 2.1. Subjects

Fifteen healthy individuals were recruited for this study. All subjects were physically active (exercising at least three times per week for more than 30 min at a time), non-smokers, free from cardiovascular and metabolic disease, not taking any medication, and weight stable for at least six months. The study was conducted according to the Declaration of Helsinki and was approved by the Loughborough University Ethical subcommittee for human participants (ethical approval number R13-P171). All subjects gave written informed consent.

### 2.2. Pre-Testing

Prior to the start of the study, subjects attended the laboratory for an initial assessment of baseline anthropometric characteristics (height, weight, and body mass index (BMI)) which were used to estimate resting energy expenditure (REE) using the calculations described by Mifflin et al. [30]. A standard correction for physical activity level (1.6 and 1.7 times REE for females and males, respectively) was applied in order to estimate total daily energy requirements. This information was then used to determine individual energy intakes for the one-day overfeeding period. 

### 2.3. Experimental Design

After the pre-testing visit, subjects attended the laboratory for an oral glucose tolerance test (OGTT) and then continued their habitual food intake for six days. On the seventh day, subjects consumed the experimental diet that was provided to them. The experimental diet was designed to be high in fat (68% total energy) and provide an energy excess (+78% kJ). High-fat foods were specifically chosen because individuals tend to overconsume more readily with high-fat foods due to the greater palatability and the higher energy density of this macronutrient, and because individuals who binge eat are known to selectively increase their intake of such foods [7]. Individual diet plans were designed using NetWISP nutrition software (Tinuviel Software Ltd., Llanfechell, Anglesey, UK). All foods were purchased and prepared by the research team. Subjects were instructed to consume all food provided, and to avoid consuming additional food or nutritive beverages. Food intake followed a normal daily feeding pattern (i.e., breakfast, lunch, dinner, and snacks) and water intake was allowed ad libitum throughout the dietary intervention. An example diet plan for one subject can be viewed in the supplementary material online. No subjects reported any issues with dietary adherence. The next day (Day 8), subjects returned to the laboratory for a second OGTT.

### 2.4. Experimental Protocol

On the experimental days (before (Day 0) and after overfeeding (Day 8)), subjects reported to the laboratory between 07.00 and 09.00 h after an overnight fast of at least 10 h and having refrained from physical activity for 48 h. After being weighed, a 20-gauge Teflon catheter (Venflon, Becton, Dickinson, Plymouth, UK) was inserted into an antecubital vein of an arm to allow for repeated blood sampling during the 2 h OGTT. A baseline (fasted (Time 0)) blood sample was taken and then subjects consumed a 25% glucose solution (75 g of glucose dissolved in 300 mL of water). Blood samples were then taken at 15, 30, 45, 60, 90, and 120 min after glucose ingestion.

### 2.5. Blood Sampling

Whole blood was collected into pre-chilled, ethylenediaminetetraacetic acid (EDTA; 1.75 mg/mL)-treated tubes (Sarstedt, Leicester, UK) and immediately spun at 1750 *g* in a refrigerated centrifuge (4 °C) for 10 min to obtain plasma, which was then stored at −20 °C until analysis. For the collection of serum, whole-blood was collected into tubes containing a clotting catalyst (Sarstedt, Leicester, UK) and left at room temperature until complete clotting had occurred. Samples were then centrifuged at 1750 *g* in a refrigerated centrifuge (4 °C) for 10 min and serum collected and stored at −20 °C until analysis.

### 2.6. Analytical Procedures

Plasma samples were analyzed using commercially available spectrophotometric assays for glucose, TG (Glucose PAP CP A11A01668, Triglycerides CP A11A01640; Horiba Medical, Northampton, UK), and NEFA (FA115; Randox, County Antrim, UK) concentrations using a semi-automatic analyzer (Pentra 400; Horiba Medical, Northampton, UK). Serum insulin concentrations were determined using an enzyme-linked immuno-sorbent assay (ELISA: EIA-2935, DRG instruments GmBH, Marburg, Germany).

### 2.7. Calculations

Plasma glucose and serum insulin concentrations obtained before and during the OGTT were used to determine whole-body insulin sensitivity using the Matsuda insulin sensitivity index (ISI):

$$\mathrm{ISI}=\frac{10,000}{\sqrt{\begin{array}{c} \left(FPG\times \;FSI\right)\times \left(mean\;OGTT\;insulin\;concentration\right)\\ \times \left(mean\;OGTT\;glucose\;concentration\right) \end{array}}}$$

where FPG is the fasting plasma glucose concentration; FSI is the fasting serum insulin concentration; and 10,000 represents a constant that allows numbers ranging between 1 and 12 to be obtained. The square root conversion is used to correct the nonlinear distribution of values [31]. Area under the curve (AUC) for glucose and insulin was calculated using the trapezoidal rule.

### 2.8. Statistics 

Data are presented as means ± standard error of the mean (SEM). Statistical analysis was performed using SPSS (V21.0) for windows (SPSS Inc., Chicago, IL, USA). Fasting concentrations of glucose, insulin, NEFA, and TG before and after high-fat overfeeding were compared using a paired *t*-test, whereas the dynamic hormonal and metabolic responses to the OGTT were compared using a two-way (trial × time) repeated measures analysis of variance (ANOVA) and Bonferroni post hoc analysis where appropriate. Statistical significance was set at *p* < 0.05.

## 3. Results

### 3.1. Diet Intervention

The estimated energy requirement, actual energy intake, macronutrient intake, and fatty acid composition of the one-day high-fat overfeeding intervention are shown in Table 1. An example diet plan for one of the subjects can be seen in the supplementary material available online.

### 3.2. Subject Characteristics

Fifteen insulin-sensitive (5.0 ± 0.5 Matsuda ISI) individuals (13 males and 2 females), with a mean BMI of 26.4 ± 1.1 kg/m^2^ were recruited (Table 2). Subjects gained on average 0.85 ± 0.20 kg body mass following one-day of high-fat overfeeding (*p* = 0.001), resulting in a BMI increase of 0.26 ± 0.06 kg/m^2^ (*p* < 0.0001) (Table 2). Whole-body insulin sensitivity (assessed by the Matsuda ISI) was reduced by 28% (*p* = 0.001) after high-fat overfeeding (Table 2). There was no change in fasting serum insulin or plasma TG concentrations following high-fat overfeeding (Table 2). Fasting plasma glucose concentrations tended to increase after high-fat overfeeding, although this did not reach significance (*p* = 0.058), whilst fasting plasma NEFA were significantly decreased (*p* = 0.009) (Table 2).

### 3.3. Oral Glucose Tolerance Test

**Figure 1:**
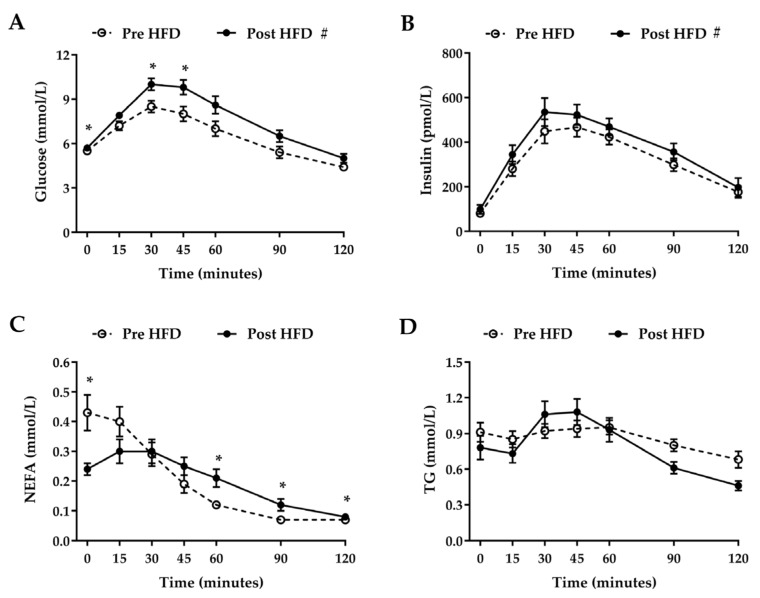
Systemic plasma glucose (**A**), serum insulin (**B**), plasma non-esterified fatty acid (NEFA) (**C**), and plasma triglyceride (TG) (**D**) concentrations during a 2-h oral glucose tolerance test (OGTT) conducted before (pre) and after (post) one day of high-fat overfeeding (HFD). Data presented are means ± standard error of the mean (SEM). *n* = 15. * *p* < 0.05 significant difference between trials at the annotated time point. # *p* < 0.05 denotes significant main effect of trial/high-fat overfeeding.

Plasma glucose and serum insulin concentrations increased in response to the OGTT, peaking 30–45 min after ingestion (Figure 1A,B). There was a significant trial × time interaction (*p* = 0.002) for plasma glucose (Figure 1A) but not for serum insulin. Postprandial plasma glucose concentrations increased by 17.1% after overfeeding (AUC, from 785 ± 35 mmol/L per 120 min before overfeeding to 920 ± 33 mmol/L per 120 min after overfeeding, *p* < 0.001). Postprandial serum insulin concentrations increased by 16.4% after overfeeding (AUC, from 39,462 ± 2840 pmol/L per 120 min before overfeeding to 45,947 ± 3396 pmol/L per 120 min after overfeeding, *p* = 0.007). A significant (*p* < 0.0001) time × trial interaction was noted for plasma NEFA (Figure 1C) despite plasma the NEFA AUC not differing between the two study days (22 ± 2 mmol/L per 120 min before overfeeding and 24 ± 2 mmol/L per 120 min after overfeeding, *p* = 0.468). A significant (*p* < 0.0001) time × trial interaction was also observed for plasma TG concentrations (Figure 1D), although average TG concentrations over the course of the postprandial period were not significantly different between study days (0.85 ± 0.06 mmol/L before overfeeding and 0.81 ± 0.07 mmol/L after overfeeding, *p* = 0.614).

## 4. Discussion

Short-term (4–14 days) adherence to a high-fat, high-energy diet has previously been reported to impair glycemic control and reduce insulin sensitivity in healthy individuals [23,24,25,27,28]. The findings we report here from our pilot study build upon this work and suggest that a single day of high-fat, high-energy food consumption impairs whole-body insulin sensitivity; evidenced by a significant (28%) reduction in whole-body insulin sensitivity as calculated by Matsuda ISI. Although our dietary model was quite extreme, with energy intake being approximately 78% greater than estimated daily requirements, individuals who demonstrate binge eating behaviors and those with BED frequently consume abnormally large amounts of food over a short period of time (i.e., within a matter of hours) [1,2,3]. Our results suggest that even brief periods of excessive consumption of foods that are typical of a Western diet may lead to metabolic dysfunction.

In the present study, participants consumed an excess energy intake as well as a high proportion of SFA. We chose this dietary intervention as the availability of palatable foods (e.g., those higher in fat) has been reported to be a precursory stimuli for binge eating [32,33] and individuals are more likely to overconsume with fat-rich foods due to the higher energy density of this macronutrient. Due to the composition of the experimental diet, we are unable to determine whether it is excess energy, excess dietary fat, or a combination of both that negatively impact glucose metabolism. Whether feeding excess energy in the form of carbohydrates (particularly added sugars, which are also highly palatable) for a single day has the same effect remains unclear. However, overfeeding a carbohydrate-rich diet (40% increase in energy intake; 60% of energy from carbohydrate) for five days was found to elicit changes in skeletal muscle cellular signaling that are typically associated with increased insulin sensitivity (i.e., increased tyrosine phosphorylation of insulin receptor-1 (IRS-1) and increased phosphatidylinositol 3 (PI 3)-kinase activity) whilst high-fat overfeeding was associated with reductions in markers of skeletal muscle insulin sensitivity (i.e., increased serine phosphorylation of IRS-1 and increased total expression of p85α) [34]. These data suggest that excessive consumption of dietary fat reduces whole-body insulin sensitivity, rather than a positive energy balance alone.

Our finding that one day of high-fat overfeeding reduces whole-body insulin sensitivity was associated with a significant (17.1%) increase in postprandial glucose AUC. Postprandial glucose homeostasis is regulated by a number of factors, including the appearance of ingested glucose, endogenous glucose production, and splanchnic and peripheral glucose uptake [35,36]. Studies in type 2 diabetic subjects demonstrate that impairments in each of these regulatory factors contribute to postprandial hyperglycemia [37,38,39,40,41,42,43,44]. As the present study was a pilot study, we measured insulin sensitivity at the whole-body level using an OGTT in combination with a validated insulin sensitivity index (the Matsuda ISI [31]). Whilst this method is easy to perform, it does not allow us to determine the contributions of each of the factors that may contribute to elevated postprandial glucose levels. Furthermore, we did not measure additional factors that may influence data, including the neural and incretin hormone response to nutrient ingestion [45,46]. It would be of interest to perform a high-fat and/or high-carbohydrate overfeeding study where whole-body and tissue-specific insulin sensitivity was assessed using clamp techniques and stable-isotope tracers [47,48,49]. Based on the observations of Brons et al. [26]**,** it is plausible that increased endogenous glucose production may underpin early diet-induced impairments in whole-body insulin sensitivity, although others suggest skeletal muscle (i.e., reduced glucose uptake) as the primary site of metabolic dysfunction [29,34,50].

In line with the discussion point above, the mechanisms underpinning the observed reduction in insulin sensitivity are yet to be elucidated. Elevated plasma NEFA concentrations have previously been implicated in the development of insulin resistance. In the present study, although fasting NEFA concentrations were reduced following high-fat overfeeding, and NEFA AUC was not significantly different between the two study days (which is in line with our previous observations following seven days of high-fat overfeeding [24]), we observed a reduction in markers of whole-body insulin sensitivity. When NEFA concentrations are elevated experimentally via Intralipid infusion, insulin sensitivity is rapidly (within 6–8 h) reduced in healthy individuals [18,19,20]. Moreover, it has been demonstrated that the ingestion of a single oral fat bolus reduced whole-body insulin sensitivity to a comparable degree, and within a corresponding time-frame (6 h post ingestion/infusion), as that seen with an energy- and composition-matched Intralipid infusion, despite divergent plasma NEFA responses [29]. The observed reduction in insulin sensitivity following both Intralipid infusion and oral fat ingestion appeared to be mediated by the increased activation of protein kinase C theta (PKCθ), which is suggested to impair insulin signaling and reduce insulin-mediated glucose uptake by inhibiting the normal tyrosine kinase cascade via phosphorylation of the counter-regulatory serine residue of insulin receptor substrate-1 (IRS-1) [51]. The reason for this apparent divergence between circulating NEFA concentrations and insulin resistance remains unclear. Whilst our findings are not directly comparable, they highlight that the relationship between circulating NEFA levels and insulin resistance is not yet fully understood.

Increased plasma TG concentrations are a characteristic feature of T2DM and the metabolic syndrome [52]. Pramfalk et al. [53] recently demonstrated that hyperinsulinemic individuals exhibit increased hepatic de novo lipogenesis and hypertriglyceridemia compared to those who were normoinsulinemic [53], which has been hypothesized to be due to an increase in the production and secretion of triglyceride-rich very low density lipoprotein (VLDL-TG) [54,55,56]. Taken together, this suggests that increased plasma TG concentrations may represent an adaptive response to hepatic insulin resistance. In the present study, we observed a reduction in insulin sensitivity but no change in fasting TG. This finding is in line with previous short-term overfeeding studies, which have observed impairments in glycemic control/reductions in insulin sensitivity alongside unchanged or even reduced fasting TG concentrations [24,25,28]. We did, however, observe a significant trial × time interaction for plasma TG across the 2-h OGTT, which appears to be due to a more dynamic/temporal postprandial response to glucose ingestion after overfeeding. Previously, it has been shown that there exists a TG storage pool within the enterocytes in which a proportion of meal-derived fatty acids are stored [57]. The amount of TG stored in this pool is related to the amount of dietary fat consumed [58]. These TGs are mobilized and secreted into the circulation following subsequent feeding, a response which has been termed “the second meal effect” [59,60,61]. Therefore, the increase in plasma TG concentration we observed following glucose ingestion after the single day of high-fat overfeeding is likely to be attributable to the second meal effect due to a larger amount of dietary TG being stored during the intervention period.

The cohort we studied were young, healthy, non-obese males and females who were recreationally active; it is therefore likely that they were relatively metabolically flexible, and somewhat able to adapt to the 1-day diet challenge. Indeed, while we saw significant increases in the postprandial glucose response after overfeeding, circulating glucose levels at 2 h post-glucose load were considerably lower than the diagnostic values of impaired glucose tolerance (i.e., 7.7–11 mmol/L) [62]. It is plausible that the dietary intervention used in the current study may produce a more dramatic effect in populations at risk of developing T2DM (e.g., sedentary, overweight individuals).

## 5. Conclusions

In conclusion, our pilot data strongly suggest that replicating excessive binge eating behavior through a single day of high-fat overfeeding is sufficient to impair whole-body insulin sensitivity in young, healthy individuals. Further research is required to elucidate the mechanisms underpinning this response and establish whether these effects persist after returning to normal eating behavior and/or whether repeated periods of binge eating leads to a progressive worsening of glycemic control. Based on our data, it is plausible to suggest that the metabolic effects of binge eating may have more marked effects in individuals at risk of insulin resistance or the metabolic syndrome. 

## Figures and Tables

**Table 1:** Energy and nutrient intakes.

	Estimated Energy Requirement	Experimental Energy Intake
Energy (kJ)	14,028 ± 433	24,949 ± 797 *
Carbohydrate (g)		192 ± 6
Protein (g)		278 ± 8
Fat (g)		449 ± 15
Fatty acid composition (%)		
SFA		42 ± 0.6
MUFA		40 ± 0.4
PUFA		10 ± 0.2

Data presented are means ± SEM. *n* = 15. * *p* < 0.05 significantly different from estimated energy requirements. Abbreviations: SFA, total saturated fatty acids; MUFA, total monounsaturated fatty acids; PUFA, total polyunsaturated fatty acids.

**Table 2:** Subject characteristics before and after one day of high-fat overfeeding.

	Before Overfeeding	After Overfeeding
Males/Females	13/2	
Age (years)	22.1 ± 0.5	
Weight (kg)	86.0 ± 3.2	86.8 ± 3.2 *
BMI (kg/m^2^)	26.4 ± 1.1	26.6 ± 1.1 *
Fasting plasma biochemical parameters and Matsuda ISI		
Glucose (mmol/L)	5.5 ± 0.1	5.7 ± 0.1
Insulin (pmol/L)	80 ± 9	98 ± 20
NEFA (mmol/L)	0.43 ± 0.06	0.24 ± 0.02 *
TG (mmol/L)	0.91 ± 0.08	0.78 ± 0.10
Matsuda ISI	5.0 ± 0.5	3.6 ± 0.4 *

Data presented are means ± SEM. *n* = 15. * *p* < 0.05 significantly different from before overfeeding. Abbreviations: BMI, body mass index; TG, triglyceride; NEFA, non-esterified fatty acids; ISI, insulin sensitivity index.

## Appendix A.

**Table:** Example diet plan for one of the subjects.

Breakfast	
Foods	Pork sausages (230 g), streaky bacon (120 g), fried eggs (180 g), fried white bread (36 g), whole milk (300 mL)
Protein (g)	86
Carbohydrate (g)	52
Fat (g)	127
Energy (kJ)	7045
% of the days intake	26
**Lunch**	
Foods	White bread (72 g), butter (15 g), cheddar cheese (70 g), mayonnaise (20 g), sausage roll (90 g)
Protein (g)	31
Carbohydrate (g)	65
Fat (g)	86
Energy (kJ)	4814
% of the days intake	17
**Snack**	
Foods	Pork pie (200 g)
Protein (g)	22
Carbohydrate (g)	47
Fat (g)	51
Energy (kJ)	3060
% of the days intake	11
**Dinner**	
Foods	Beef burgers (300 g), streaky bacon (120 g), cheddar cheese (90 g), coleslaw (150 g)
Protein (g)	95
Carbohydrate (g)	7
Fat (g)	173
Energy (kJ)	8135
% of the days intake	30
**Dessert**	
Foods	Chocolate chip muffin (70 g), double cream (150 mL)
Protein (g)	6
Carbohydrate (g)	37
Fat (g)	98
Energy (kJ)	4357
% of the days intake	16
**Total intake**	
Protein (g)	240
Carbohydrate (g)	209
Fat (g)	535
Energy (kJ)	27,411

Water intake was allowed ad libitum throughout the day.
